# Pornography use and sexting amongst children and young people: a systematic overview of reviews

**DOI:** 10.1186/s13643-020-01541-0

**Published:** 2020-12-06

**Authors:** Gary Raine, Claire Khouja, Rachel Scott, Kath Wright, Amanda J. Sowden

**Affiliations:** 1 Centre for Reviews and Dissemination, University of York, Second floor, Alcuin B Block, York, YO10 5DD UK; 2 London School of Hygiene and Tropical Medicine, Room 148, Keppel Street, London, WC1E 7HT UK

**Keywords:** Pornography, Sexting, Children, Young people, Review of reviews, Overview, Systematic review

## Abstract

**Background:**

Young people’s use of pornography and participation in sexting are commonly viewed as harmful behaviours. This paper reports findings from a ‘review of reviews’, which aimed to systematically identify and synthesise the evidence on pornography and sexting amongst young people. Here, we focus specifically on the evidence relating to young people’s use of pornography; involvement in sexting; and their beliefs, attitudes, behaviours and wellbeing to better understand potential harms and benefits, and identify where future research is required.

**Methods:**

We searched five health and social science databases; searches for grey literature were also performed. Review quality was assessed and findings synthesised narratively.

**Results:**

Eleven reviews of quantitative and/or qualitative studies were included. A relationship was identified between pornography use and more permissive sexual attitudes. An association between pornography use and stronger gender-stereotypical sexual beliefs was also reported, but not consistently. Similarly, inconsistent evidence of an association between pornography use and sexting and sexual behaviour was identified. Pornography use has been associated with various forms of sexual violence, aggression and harassment, but the relationship appears complex. Girls, in particular, may experience coercion and pressure to engage in sexting and suffer more negative consequences than boys if sexts become public. Positive aspects to sexting were reported, particularly in relation to young people’s personal relationships.

**Conclusions:**

We identified evidence from reviews of varying quality that linked pornography use and sexting amongst young people to specific beliefs, attitudes and behaviours. However, evidence was often inconsistent and mostly derived from observational studies using a cross-sectional design, which precludes establishing any causal relationship. Other methodological limitations and evidence gaps were identified. More rigorous quantitative studies and greater use of qualitative methods are required.

**Supplementary Information:**

The online version contains supplementary material available at 10.1186/s13643-020-01541-0.

## Background

Over the last decade, there have been multiple independent reviews conducted on behalf of the UK government into the sexualisation of childhood and the safety of young people online and on other digital media (for example, Byron [1]; Papadopoulos [2]; Bailey [3]). Similar reports have also been published in other countries including Australia [4–6]; France [7]; and the USA [8]. On the basis of a presumed need to protect children from sexually explicit material online, the UK government included in the Digital Economy Act [9], a requirement for pornographic websites to implement age verification checks. However, following several delays in implementation, it was announced in autumn 2019 that checks would not be introduced [10]. Instead, the objectives of the Digital Economy Act in relation to preventing children’s exposure to online pornography are to be met through a new regulatory framework set out in the Online Harms White paper [11]. This White paper proposes establishing a statutory duty of care on relevant companies to improve online safety and tackle harmful activity, which will be enforced by an independent regulator [11].

It has often been suggested that children and young people’s viewing of pornography leads to harm (for example, Flood [12]; Dines [13]). In addition, sexting (a portmanteau of ‘sex’ and ‘texting’) is often framed within a discourse of deviance and the activity viewed as a high-risk behaviour for young people [14]. Some suggested harms include sexual violence and coercion to engage in sex-related activities, although what is meant by harm has not always been clearly articulated.

This paper reports findings from a ‘review of reviews’ commissioned by the Department of Health and Social Care (DHSC) in England, which aimed to systematically identify and synthesise the evidence on pornography and sexting amongst children and young people. Given the wide scope, a ‘review of reviews’ (RoR) was considered the most appropriate method. RoRs identify, appraise and synthesise findings from existing reviews in a transparent way and can also highlight the absence of evidence [15–19]. Here, we focus specifically on the evidence relating to young people’s use of pornography; involvement in sexting; and their beliefs, attitudes, behaviours and wellbeing, to better understand potential harms and benefits, and to identify where future research is required.

## Method

We searched five electronic databases using a range of topic terms and synonyms, including “pornography”, “sexually explicit content” and “sexting”, combined with a search filter for systematic reviews^1^. The full search strategy is available as a supplementary file (Additional file 1). The following databases were searched up to August/September 2018: Applied Social Science Index & Abstracts (ASSIA), MEDLINE and MEDLINE in Process, PsycINFO, Scopus and Social Science Citation Index. No restrictions were placed on date of publication or geographical location. In addition, supplementary searches were conducted of the websites of key organisations, including the Children’s Commissioner for England; the National Society for the Care and Protection of Children (NSPCC) and the website of the UK government. We searched for other grey literature using the advanced search function of Google.

The title and abstract of records, and full-text papers were screened by two reviewers independently. Findings reported in the current paper were based on reviews meeting the following criteria:

- Focused on children and young people’s (however defined) use of pornography, sexting or both. Any type of pornography (printed or visual) was considered relevant.
- Reported findings related to pornography and sexting and their relationship to young people’s beliefs, attitudes, behaviours or wellbeing.
- Used systematic review methods, which required authors to have, as a minimum: searched at least two sources, one of which must have been a named database; clear inclusion/exclusion criteria covering key review components; and provided a synthesis of findings. This could be a statistical synthesis in the form of a meta-analysis or a narrative synthesis of findings from included studies. Reviews were not eligible for inclusion if authors simply described each individual included study with no attempt made to bring together findings on the same outcome from multiple studies.

Reviews needed to have a main focus on pornography or sexting and young people and could include primary studies of any design (quantitative and/or qualitative). Reviews were excluded if they focused primarily on sexually explicit content in non-pornographic popular media such as television programmes, video games or music videos. Sexting was conceptualised broadly as sending or receiving sexually explicit photographs or messages via a mobile phone or other media devices.

Data were extracted from each review on key characteristics including review methods, population(s) and outcomes. Data extraction was conducted by one reviewer and checked by a second reviewer.

Each review was critically appraised according to modified Database of Abstracts of Reviews of Effects (DARE) criteria [20]. Review quality was assessed by one reviewer and checked by another. The critical appraisal process was used to inform judgements about potential sources of bias and threats to the validity and reliability of findings reported across reviews.

Findings were synthesised narratively across reviews and compared and contrasted, where appropriate. During the synthesis process, all data extracted from reviews relating to the same broad category or theme (for example sexual behaviour, sexual attitudes) were brought together and similarities and differences in findings identified both across reviews and across studies within reviews. A descriptive summary of the main findings reported in the reviews was then produced. Findings from quantitative and qualitative studies were synthesised separately under the relevant topic heading. We made no assumptions during the synthesis process about whether specific outcomes are harmful or not. The term young people is used in the following section to cover both young people and children. We did not register a protocol for this review on PROSPERO due to time constraints, but we did produce a project brief which was approved by DHSC. This set out the focus for the review, methods to be used and a timetable for the work.

## Results

**Fig. 1:**
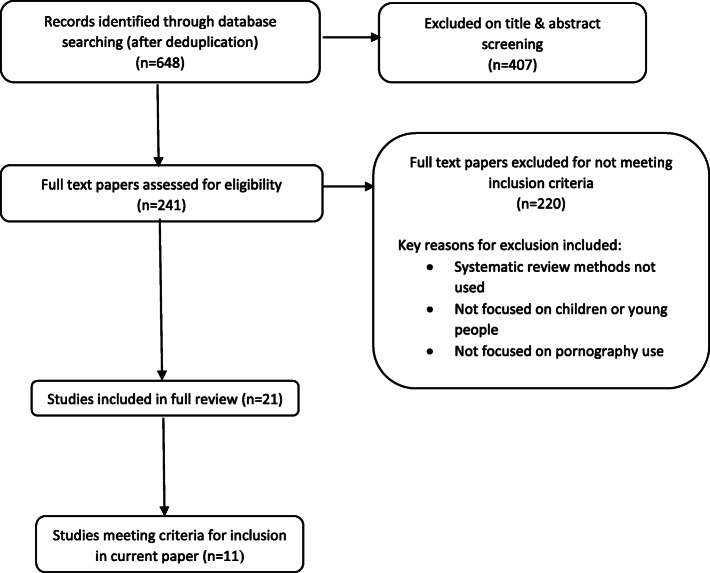
Flow of studies through the review

After deduplication, 648 titles and abstracts and 241 full-text papers were screened. Eleven reviews met the inclusion criteria stated above. The flow of the literature through the review is shown in Fig. 1.

### Description of reviews

Of the 11 reviews, three focused on pornography [21–23]; seven focused on sexting^2^ [24–30]; and one review addressed both pornography and sexting [31]. Key characteristics of the 11 reviews are provided in Table 1.

**Table 1:** Included reviews

First author	Main synthesis of findings	Focus	Search dates	Number of included studies^b^	Publication dates of included studies	Age range or mean age (years)
1. Anastassiou (2017) [27]	Narrative	Sexting	NR	8	2012–2016	12–25
2. Barrense-Dias (2017) [28]	Narrative	Sexting	No date restriction-search conducted Nov 2015	18	2012–2015	10–18
3. Cooper (2016) [25]	Narrative	Sexting	2009–Sept 2014	88	Unclear	Under 25
4. Handschuh (2019) [30]	Meta-analysis	Sexting	Up to April 2017	9 (6 in meta-analysis)	2012–2015 (in meta-analysis)	10–19
5. Horvath (2013) [21]	Narrative	Porn	1983–Jan 2013	159	1992–2013	Up to 18
6. Koletić (2017) [23]	Narrative	Porn	NR-search conducted in Sept 2015	9 studies (20 papers)	2008–2015	Mean age: under 18
7. Kosenko (2017) [29]	Meta-analysis	Sexting	No date restriction	15	2011–2015	10–51^c^
8. Peter (2016) [22]	Narrative	Porn	1995–Dec 2015	75	1995–2015	Mean age: 10–17
9. Van Ouytsel (2015) [24]	Narrative	Sexting	2008–March 2014	9	2012–2014	10–20 (inclusion criteria 10–21)
10. Watchirs Smith (2016) [31]	Meta-analysis	Porn and sexting	2005–May 2014	14 (6 porn; 8 sexting)	2005–2012 (porn) 2011–2014 (sexting)	10–24
11. Wilkinson (2016) [26]	Qualitative meta-synthesis^a^	Sexting	Up to Nov 2015	5	2009–2013	1 study: 18–30 years Others: 11–20 years

*NR* not reported; ^a^‘Qualitative meta-synthesis’ was a term used by the review authors. ^b^Not all included studies reported findings relevant to the current RoR. ^c^Only one study in the Kosenko et al. review included any participants over the age of 30, and these were considered outliers as the mean age of the sample was 21 years old. Separate analyses were conducted to partially account for age, but no statistically significant differences in effect sizes were reported between teenagers and older participants

Two reviews reported qualitative findings only [26, 27]. Five reviews reported quantitative findings only [23, 24, 29–31], and four reported findings from both types of primary study [21, 22, 25, 28]. One review reported solely on findings from longitudinal studies [23]. Eight reviews included either cross-sectional studies only or both cross-sectional and longitudinal research [21, 22, 24, 25, 28–31]. Across reviews, most studies were cross-sectional and data collected using methods such as questionnaire-based surveys, one-to-one interviews and focus groups.

Data in three reviews were synthesised statistically using meta-analysis [29–31] and one review conducted a meta-ethnographic qualitative synthesis [26]. Other reviews reported a narrative synthesis of findings. Across the reviews, most included studies appeared to originate from the USA and Europe (mainly the Netherlands, Sweden and Belgium), but information about country of origin was not reported systematically.

Overall, included reviews with the same topic focus were similar in terms of scope and inclusion criteria. The publication dates of included studies in eight of the 11 reviews ranged between 2008 and 2016 [23, 24, 26–31]. The population of interest for every review included children ranging in age from pre-teens to 18 years, but there was variation between reviews in terms of the upper age limit, which is discussed further in the limitations section. Other differences between reviews were noted: In terms of pornography, Watchirs Smith et al. [31] focused on exposure to content on sexually explicit websites/internet-based pornography. In addition, both Handschuh et al. [30] and Cooper et al. [25] focused on sending sexts as opposed to receiving them.

Horvath et al. [21] described their review as a ‘rapid evidence assessment’ and included not only academic and non-academic primary research but also ‘reviews’ and meta-analyses, policy documents and other ‘reports’. Similarly, the eligibility criteria used by Cooper et al. [25] allowed for the inclusion of ‘non-empirical research discussions’ (p.707) as well as primary studies. Across reviews, several publications were linked to the same research study. For example, Koletić [23] included 20 papers that were linked to nine different research studies. In addition, Peter and Valkenburg [22] reported that multiple studies/papers had used the same data sample.

There was considerable overlap in the primary studies included across reviews, which was not unexpected given the similarity in scope between reviews. For example, three reviews synthesised narratively quantitative data on the relationships between sexting and sexual behaviour, and between sexting and non-sexual health risk behaviour such as substance use. Barrense-Dias et al. [28] cited seven different papers that addressed these relationships, Van Ouytsel et al. [24] cited five, and three papers were common to both reviews. All five of the papers cited by Van Ouytsel et al. and four by Barrense-Dias et al. were also included by Cooper et al. [25]. Reviews by Horvath et al. [21], Peter and Valkenburg [22] and Koletić [23] had four studies in common that addressed pornography use and permissive attitudes and gender-stereotypical sexual beliefs.

### Review quality

Assessments of the reviews against the modified DARE criteria are shown in Table 2. All reviews were rated as being adequate for scope of literature searching and reporting of inclusion/exclusion criteria. In nine reviews, searches were conducted of at least three databases [21, 23–26, 28–31]. In two reviews, searches were conducted using a smaller number of databases, but were supplemented by using other sources such as reference list checking or internet searching [22, 27]. In two reviews, only the single word, ‘sexting’ was used as a search term [24, 29]. All reviews reported eligibility criteria covering all or most of the following key review components: population; behaviour (i.e. pornography, sexting or both); issue or outcomes of interest; and publication/study type.

**Table 2:** Critical appraisal of included reviews based on modified DARE criteria

Critical appraisal questions	Anastassiou (2017) [27]	Barrense-Dias (2017) [28]	Cooper (2016) [25]	Handschuh (2019) [30]	Horvath (2013) [21]	Koletić (2017) [23]	Kosenko (2017) [29]	Peter (2016) [22]	Van Ouytsel (2015) [24]	Watchirs Smith (2016) [31]	Wilkinson (2016) [26]
1. Was an adequate search conducted?^a^	Yes	Yes+	Yes+	Yes+	Yes+	Yes+	Yes+	Yes	Yes+	Yes+	Yes+
2. Was there adequate reporting of inclusion/exclusion criteria?^b^	Yes	Yes	Yes	Yes	Yes	Yes	Yes	Yes	Yes	Yes	Yes
3. Were data synthesised?^c^	Yes	Yes+	Yes	Yes+	Yes	Yes	Yes+	Yes+	Yes+	Yes+	Yes+
4. Was the quality of individual studies assessed?	Yes	No	No	Yes	Yes	No	No	No^e^	No	Yes	Unclear^e^
5. Were adequate study details reported?^d^	No	Yes	No	Yes	No	Yes	Yes	Yes	Yes	Yes	Yes

^a^Yes = Reported a search of up to two databases plus at least one other source; Yes+ = Searched at least three databases. ^b^Yes = Reported criteria covering all or most of the following key review components: population; behaviour (i.e. pornography, sexting or both); issue or outcomes of interest; and publication/study type. ^c^Yes = Adequate narrative synthesis reported. Yes+ = Data from multiple studies combined statistically using a well-described process of meta-analysis or authors provided a more detailed and comprehensive narrative synthesis. ^d^Yes = Review included a table of characteristics that reported a range of relevant information about each included study. No = Few details about included studies were reported. ^e^further information provided in the main text

The extent to which authors synthesised findings was variable but adequate in all reviews. Three of the reviews that synthesised results narratively were rated higher on this criterion as they provided a synthesis that was more detailed and comprehensive in drawing together and reporting findings from multiple studies [22, 24, 28].

Reviews were also assessed according to two additional criteria: the reporting of study details, and whether an evaluation of the methodological quality of included studies was reported. Eight reviews provided details of included studies in the form of a table of characteristics that reported a range of relevant information about the population sample, study design, variables and/or outcomes of interest/key findings [22–24, 26, 28–31]. The other three reviews provided few details about included studies [21, 25, 27].

In four reviews, some form of quality assessment was reported [21, 27, 30, 31]. In addition, Peter and Valkenburg [22] did not conduct a quality assessment of individual studies, but they did report a critical evaluation of findings from their review, which included identifying bias from study designs and sampling methods. Wilkinson et al. [26] reported excluding papers on the basis of low methodological quality but did not explicitly state that a quality assessment had been conducted. Horvath et al. [21] reported placing less emphasis in the synthesis on studies rated as ‘lower quality’ based on a modified ‘Weight of Evidence’ assessment [32].

It can be seen from Table 2 that two reviews (Handschuh et al. [30] and Watchirs Smith et al. [31]) were assessed as meeting all five criteria. Five reviews (Van Ouytsel et al. [24]; Peter and Valkenburg [22]; Barrense-Dias et al. [28]; Kosenko et al. [29] and Wilkinson [26]) met four criteria, including reporting a higher quality narrative synthesis of findings or a meta-analysis.

The reporting of review methods was generally inadequate across all reviews, which precluded an assessment of overall reliability or potential for bias. For example, most of the reviews did not provide information about the number of reviewers involved in screening decisions or data extraction.

### Sexual attitudes and beliefs

Evidence was consistent across four reviews for a relationship between young people’s viewing of sexually explicit material, and stronger permissive sexual attitudes [21–23, 31]. ‘Permissive sexual attitudes’ is a term used across reviews, but not always defined. Peter and Valkenburg [22] used it to describe positive attitudes towards casual sex, typically outside of a romantic relationship.

Four reviews reported evidence of an association between pornography use and stronger gender-stereotypical sexual beliefs, including viewing women as sex objects, and less progressive attitudes to gender roles [21–23, 31]. However, evidence for a relationship between pornography and gender-stereotypical sexual beliefs was not consistently identified. One longitudinal study included in three reviews found no association between frequency of viewing internet pornography and gender-stereotypical sexual beliefs [21–23].

Evidence was reported across three reviews suggesting a relationship between pornography use and a range of other sexual attitudes and beliefs, including sexual uncertainty; sexual preoccupancy; sexual satisfaction/dissatisfaction; unrealistic beliefs/attitudes about sex and ‘maladaptive’ attitudes towards relationships [21–23]. These findings were often based on one or two studies only, with overlap across reviews.

### Sexual activity and sexual practices

Evidence from longitudinal and cross-sectional studies reported in four reviews suggested an association between pornography use and an increased likelihood of engaging in sexual intercourse and other sexual practices such as oral or anal sex [21–23, 31]. Gender and pubertal status were identified as moderators of the association between pornography use and initiating sexual intercourse in one review [22]. Studies were also reported across reviews that did not find a relationship between pornography use and various types of sexual practices and behaviour, including intercourse before the age of 15, or studies found associations that were inconsistent [21–23, 31].

An association between pornography use and engaging in casual sex or sex with multiple partners was reported in three reviews [21, 22, 31]. However, an association between casual sex and pornography use was only found for female adolescents in one of the studies included by Peter and Valkenburg [22]. In addition, one study reported across three reviews did not find a significant association between pornography use and having a higher number of sexual partners [21, 22, 31].

Evidence linking pornography use to sexual risk taking in young people was inconsistent. Three reviews reported an association between pornography use and ‘risky’ sexual behaviour, including having unprotected sex and using drugs/alcohol during sex [21, 22, 31]. However, another study included in two reviews failed to identify an association between pornography use and engaging in unprotected casual sex [22, 23].

Both Horvath et al. [21] and Peter and Valkenburg [22] included qualitative studies that suggested young people may learn sexual practices and scripts for sexual performance from pornography, which can influence their expectations and behaviour. Pornography was also seen as a standard by which to judge sexual performance and body ideals in some qualitative studies. Evidence reported by Horvath et al. [21] indicated that some young people saw pornography as a positive source of sexual knowledge, ideas, skills and confidence.

An association between sexting and engaging in various types of sexual activity was identified in six reviews [24, 25, 28–31]. A recent meta-analysis of six studies [30] found that the odds of reporting either past or current sexual activity were approximately six times higher for young people who sent sexts, compared with those who did not (OR 6.3, 95% CI: 4.9 to 8.1). An earlier meta-analysis [31] found that sexting was associated with an increased likelihood of ever having had sex (vaginal only or vaginal, anal or oral) (OR 5.58, 95% CI: 4.46 to 6.71, five studies) as well as with recent sexual activity (OR 4.79, 95% CI: 3.55 to 6.04, two studies). Another meta-analysis of 10 studies [29], reported an association between sexting and engaging in ‘general sexual activity’ (*r* = 0.35, 95% CI: 0.23 to 0.46). There was notable overlap in the primary studies across the meta-analyses by Watchirs Smith et al. [31], Kosenko et al. [29] and Handschuh et al. [30]. Five out of the 10 studies included in the meta-analysis by Kosenko et al. had been included in the earlier meta-analysis by Watchirs Smith et al. that was focused on having ‘ever’ engaged in intercourse. The most recent meta-analysis by Handschuh et al. included only one study that was not in the meta-analysis by Kosenko et al. In addition, the same three studies were included in all three meta-analyses.

Four reviews identified an association between sexting and having a higher number of sexual partners [29] or multiple partners, over varying time periods [24, 25, 31]. However, in one of the studies reported by Van Ouytsel et al. [24] an association was only present amongst girls. Kosenko et al. [29] reported that the association between sexting and number of partners was small (*r* = 0.20, 95% CI: 0.16 to 0.23, seven studies). Watchirs Smith et al. [31] found that the likelihood of having multiple sexual partners in the past 3 to 12 months was approximately three times higher amongst young people who sexted compared with those who did not (OR 2.79, 95% CI: 1.95 to 3.63; two studies).

Inconsistent evidence for an association between sexting and ‘risky’ sexual behaviours was reported across five reviews [24, 25, 28, 29, 31]. Kosenko et al. [29] found an association between sexting and engaging in unprotected sexual activity from a pooled analysis of nine studies, but the size of the relationship was small (*r* = 0.16, 95% CI: 0.09 to 0.23). In contrast, another meta-analysis of two studies [31] found no association between sexting and engaging in condomless anal intercourse in the past one or two months (OR 1.53, 95% CI: 0.81 to 2.25). Three reviews [24, 25, 31] reported that sexting was associated with the use of alcohol or other drugs before/during sex (Watchirs Smith, OR 2.65, 95% CI: 1.99 to 3.32; two studies) [31].

### Other risk behaviours

An association between sexting and substance use (alcohol, tobacco, marijuana and other illicit drugs) was reported in three reviews [24, 25, 28]. In addition, a single study reported by Barrense-Dias et al. [28] found an association between sexting and physical fighting amongst boys. The same authors also identified evidence from another study of a relationship between sexting and other ‘risky’ behaviours such as truancy and getting into trouble with teachers or the police. Similarly, one study included by Van Ouytsel et al. [24] reported that school students who sexted were more likely to have engaged in ‘delinquency’. The variable ‘delinquency’ was defined by respondents’ previous engagement in nine behaviours that the study authors viewed as delinquent activities, such as stealing, truancy, smoking and drinking. Evidence of a link between pornography and rule breaking or delinquent behaviour was reported in two reviews [21, 22]. Furthermore, both Horvath et al. [21] and Peter and Valkenburg [22] included the same single study that identified an association between pornography and substance use.

### Sexual violence and aggression

An association between exposure to sexually explicit media and various forms of sexual violence and aggression has been found in both longitudinal and cross-sectional research. Three reviews identified an association between pornography use and the perpetration of sexual harassment or sexually aggressive behaviour, including forced sexual activity [21–23]. In one study reported across the three reviews, a link between sexual harassment perpetration and viewing sexually explicit media was found for boys only. Another study included by Horvath et al. [21] reported findings suggesting that pornography was only associated with sexual violence in young men who had a predisposition for aggressive sexual behaviour. Furthermore, a longitudinal study included in all three reviews found an association between pornography use and sexual aggression or assault, but only when violent material was viewed. Peter and Valkenburg [22] also reported evidence from one study that found an association between sexual violence or harassment and the use of pornographic magazines and comics, but identified no association with the use of pornographic films and videos. In two studies reviewed by Horvath et al. [21], the frequent use of pornography and/or watching violent pornography were more common amongst male and female high school students who had engaged in sexually coercive behaviour compared with peers who had not.

Two reviews reported an association between viewing pornography and being a victim of sexual violence or sexual harassment, especially amongst young women [21, 22]. Three reviews reported findings from one study that found sexting adolescents were more likely to ever have been forced to have sex, and to have been subjected to physical violence by their partner in the previous year, than adolescents who had not engaged in sexting [24, 25, 31]. Cooper et al. [25] further reported an association between receiving a sext and experiencing interpersonal violence from a single study of university students.

### Coercion, bullying and harassment

Three reviews reported that girls, in particular, may experience coercion and pressure to engage in sexting [25, 26, 28]. An association was also identified between bullying, cyberbullying or harassment and sexting [24, 25, 28]. For example, one cross-sectional study included by Barrense-Dias et al. [28] found that adolescent girls who had been a victim of cyberbullying were more likely to sext. Furthermore, Cooper et al. [25] identified a greater risk of various types of cyber victimisation for females who engaged in sexting based on one cross-sectional study of college students. They also reported findings from another study which suggested that young people who voluntarily engaged in ‘sexual exposures’ on the internet were more likely to both receive and perpetrate online harassment.

Qualitative findings reported in four reviews suggested that girls who engaged in sexting may receive more negative treatment than boys, and also potentially experience greater judgement and reputational consequences, if images become public as a result of non-consensual sharing [25–28]. One quantitative study reviewed by Cooper et al. [25] found that boys, in particular, were likely to experience bullying or be the victims of non-consensual sharing of images. Both Cooper et al. [25] and Handschuh et al. [30] also reported that females were more bothered by requests to sext than males.

### Mental health and wellbeing

Single studies reported by Koletić [23] and Peter and Valkenburg [22] linked the use of pornography to increased body surveillance in boys. In addition, Horvath et al. [21] and Peter and Valkenburg [22] included qualitative studies which found that young women, in particular, believed that pornography portrayed an unattainable female body ideal, and they felt unattractive in comparison. They also reported feeling pressured by the messages related to body image conveyed by pornography. Horvath et al. [21] reported inconsistent evidence of an association between pornography and depression: exposure to pornography was related to depression in two studies, but a third found no association between accessing pornographic material and depression or loneliness. Koletić [23] reported findings from a longitudinal study that found depression at baseline was associated with the compulsive use of pornography by adolescents 6 months later.

Three reviews reported inconsistent evidence on the relationship between sexting and mental health [24, 25, 28]. One study included by Barrense-Dias et al. [28] identified an association between ‘psychological difficulties’ and an increased likelihood of receiving sexts and being ‘harmed’ by them. All three reviews reported evidence of a relationship between depression, or depressive symptoms and sexting. In a single study included by both Van Ouytsel et al. [24] and Cooper et al. [25], an association was reported between engaging in sexting and feeling sad or hopeless for more than two weeks in the previous year. An association was also identified between sexting and having contemplated or attempted suicide in the previous year. In one study reviewed by Barrense-Dias et al. [28], an association with depression was only identified for younger females. Other studies reported across the three reviews found no relationship between sexting and depression, or sexting and anxiety [24, 25, 28].

In one survey of 1,560 youth internet users included in three reviews, a fifth of respondents who sent a sext reported a negative emotional effect (feeling very or extremely upset, embarrassed or afraid) [24, 25, 28]. Also based on the findings from a single study, Barrense-Dias et al. [28] suggested that girls and younger adolescents were more likely to report upset or harm from sexting.

### Relationships

Three reviews identified positive aspects to sexting in relation to the personal relationships of young people [25–27]. For example, sexting has been described by some young people as a safe medium for flirting and experimentation, as well as a safer alternative to having sex in real life. Sexting was also reported to help maintain long-distance relationships.

## Discussion

The findings from 11 reviews were synthesised to provide an overview and assessment of the current evidence in relation to young people’s use of pornography and involvement in sexting, and their beliefs, attitudes, behaviour and wellbeing. Studies on both pornography and sexting have often been framed within a ‘negative effects’ paradigm, which assumes specific sexual behaviours represent inherent risks or harms [33]. In this paradigm, exposure to sexually explicit media is considered a potential stimulus to engagement in ‘harmful’ behaviours [33, 34].

This RoR identified an association between both pornography use and sexting and certain sexual behaviours. Some of these behaviours, such as engaging in casual sex, anal sex or having a higher number of partners, may in certain circumstances carry some risks, but none of them, nor holding permissive sexual attitudes, are in themselves inherently harmful [33, 35].

Evidence of an association between sexual behaviours and pornography use, in particular, was often inconsistent across reviews and across studies within reviews. Inconsistent findings were also reported on the relationship between both pornography and sexting and mental health, as well as between pornography use and gender-stereotypical sexual beliefs. The relationship between pornography use and sexual violence and aggression appears complex with some studies suggesting an association only with certain sources of pornography, specific pornographic content or for young men who are prone to aggressive behaviour.

### Methodological issues

Review quality varied and most had some key limitations, but all eleven were considered to be of an adequate standard. Notably, reviews by Horvath et al. [21] and Cooper et al. [25] potentially included evidence from an unknown number of non-empirical publications. Given the uncertainty regarding the sources of evidence presented in these two reviews, their findings should be treated with caution.

Other key methodological issues were identified with reviews and the primary studies included in them. Importantly, most of the evidence on pornography and sexting is derived from observational studies using a cross-sectional design. This means it is not possible to draw conclusions about whether reported associations are a consequence or a cause of viewing pornography or engaging in sexting. For example, it could be the case that sexting encourages young people to engage in sexual activity. However, as Kosenko et al. [29] pointed out, it is equally likely that sexting is simply an activity performed by individuals who are already sexually active, and the same also holds true with regard to the viewing of pornography. Similarly, individuals who already have stronger permissive attitudes and gender-stereotypical beliefs may be more drawn to pornography.

Review authors cited the cross-sectional nature of the evidence as a significant limitation, and more prospective longitudinal research was suggested to improve understanding of the temporal relationship between pornography or sexting and a range of outcomes. Peter and Valkenburg [22] emphasised the need to include a range of potentially significant control variables in statistical analyses of longitudinal data to reduce the likelihood of confounding and obtaining spurious associations. Importantly, these authors also highlighted the fact that whilst longitudinal studies generally have greater methodological rigour than cross-sectional designs, they are still correlational in nature and do not demonstrate causality.

Given the potential for spurious associations due to confounding, findings from existing studies should be treated with caution. Peter and Valkenburg [22] highlighted wide variation in the extent to which researchers had attempted to adjust for confounding in existing studies, with some only controlling for a limited number of variables such as individual demographics. It is likely that recognised predictors of behaviour and other potentially important confounding variables may not have been controlled for during analyses, which limits the degree of confidence that can be placed in findings.

Evidence suggests that insufficient attention has been given to contextual factors in quantitative studies on sexting and young people. For example, none of the studies reviewed by Van Ouytsel et al. [24] had distinguished between the different contexts in which sexting may occur, and this was recognised to be a potential limitation. Sexting-related outcomes could be influenced by a number of different contextual factors including the relationship status of the individuals involved and their motives for sexting. Van Ouytsel et al. suggested that some of the reported associations between sexting and behaviour may not hold true after controlling for the context in which sexting occurred.

Similar studies reported inconsistent findings on the relationship between pornography and sexting and multiple outcomes of interest. Inconsistency is likely to be related, at least in part, to heterogeneity in how previous research has been operationalised. In particular, there was marked variation in the conceptualisation and definition of both sexting and pornography. For example, multiple sexting reviews [28–31] reported that studies varied in whether the focus was on messages being sent, received or both. Differences were also noted in the types of messages studied, (such as image only, text and images or video), and in the terminology used to describe message content, with terms being open to individual interpretation. For example, terms included ‘sexy’, ‘sexual’ ‘sexually explicit’, ‘suggestive’, ‘provocative’, ‘erotic’ ‘nearly nude’ or ‘semi-nude’. Similarly, differing definitions and terminology have been used in pornography studies, for example ‘X-rated material’; ‘sexually explicit media’; and ‘sexualised media’ [23]. Such differences were seen to reflect variation between studies in the conceptualisation of pornography and specific content of interest. Review authors highlighted a failure in some studies to provide a definition or explanation of key terms. Variability was also found in other important factors such as age range, specific outcomes studied, outcome measurement and recall periods for behaviour (e.g. ever, within the last year or last 30 days). Together, these factors make comparisons between study findings, and assessing the overall evidence base, extremely difficult.

The problem of heterogeneity was highlighted in the three reviews using meta-analysis. Watchirs Smith et al. [31] stated that a pooled estimate was not calculated for the association between pornography use and sexting and several forms of sexual activity due to high statistical heterogeneity. In addition, both Kosenko et al. [29] and Handschuh et al. [30] reported substantial levels of heterogeneity in their pooled analyses. Handschuh et al. [30] reported multiple meta-analyses related to sexting and sexual activity: findings were reported for all adolescents combined, and then for males and females separately. Analyses revealed heterogeneity to be greater than expected by chance alone, with *I*^2^ estimated at 65% for all adolescents. Values for *I*^2^ of 50% and 75% are considered to represent moderate and high heterogeneity respectively [36]. When analysed by sex, very high levels of heterogeneity were found: *I*^2^ = 86.4% for males and *I*^2^ = 95.8% for females. Subgroup analyses were conducted, but could not explain the heterogeneity. Kosenko et al. [29] also reported analyses for various types of sexual activity and sexting in which heterogeneity was calculated to be *I*^2^ = 98.5% (general sexual activity); *I*^2^ = 87.5% (unprotected sex) and *I*^2^ = 42.7% (number of sex partners). Given the high levels of heterogeneity found, findings should be treated with caution.

It was not possible to assess the extent of study overlap in reviews for all reported outcomes. However, as expected, we found that for some outcomes there was considerable overlap in the studies included across reviews and in meta-analyses. This included overlap in studies reporting on the association between pornography use and sexual beliefs, attitudes and activity and between sexual activity and engaging in sexting. The inclusion of the same study or studies in multiple reviews may offer some reassurance that individual reviews have been conducted in a consistent manner and their results reflect the available literature. However, the presence of overlapping primary studies in reviews is recognised to be a potential issue for RoRs [16, 18]. For example, study overlap can be a potential source of bias, when specific studies, particularly those that are small or of poorer quality, become over-represented through their inclusion in multiple reviews [16]. It may also lead to an overestimation of the size and strength of the evidence base.

### Key evidence gaps and future research

The term pornography covers an array of different material and the type of content watched may be important in terms of potential harms, as indicated by the findings on the relationship between violence and pornography (i.e. a link with aggression was identified only when violent pornography was viewed). Whilst some research has focused on specific sources of material, such as online pornography, studies with young people appear to have largely treated pornography as a homogenous entity in terms of content. As some authors have identified, there is a need for more research that investigates separately, or disaggregates the effects of, different types of pornographic content [23].

Whilst there is concern that many young people are accessing highly stylised, degrading or violent pornography, there also exists a general lack of knowledge and understanding about what pornographic material young people are actually viewing [21, 22]. Current discourse is based largely on opinion or speculation about what young people are accessing [21]. More research is required to investigate the type of pornographic content that young people are viewing rather than relying on speculation.

Evidence was identified to suggest that young people are not uncritically accepting of what they see in pornographic material. For example, Peter and Valkenburg [22] indicated that on average young people did not view pornography as a realistic source of sexual information. Similarly, Horvath et al. [21] reported evidence that many young people recognised that pornography may portray distorted messages about sexual activity, relationships, power and body ideals. Such findings are consistent with other media research, which indicated that young people are not simply passive ‘dupes’ or ‘victims’ of media messages. Instead, young people were found to adopt a critical and active role in interpreting various media [37–40].

Various authors including Attwood [34] and Horvath et al. [21] have highlighted the value of conducting more research focused on the ways in which young people actually view, understand and engage with various forms of explicit media. Further qualitative research that explores the factors that influence young people’s perceptions of pornography, and their reactions to it, may be particularly informative.

Non-consensual forwarding of sexts was identified as a significant concern. Potential negative consequences for the sender were reported if sexts were made public, which included reputational damage, harassment and cyberbullying. However, it is important to recognise that such consequences are not a direct or inevitable outcome of sending a sext. Rather they result from a betrayal of trust as well as from victim blaming and gendered cultural norms related to what is acceptable sexual behaviour and self-representation, particularly for girls [14, 41]. Qualitative studies suggest that the non-consensual sharing of sexts most commonly affects girls, but this is not supported by existing quantitative data. A meta-analysis conducted by Madigan et al. [42] found no association between sex/gender and the prevalence of either having a sext forwarded without consent or perpetrating non-consensual sexting. The authors cautioned that the meta-analyses on the non-consensual sharing of sexts were based on small sample sizes and recommended additional research to examine prevalence. In addition to further quantitative studies, the non-consensual forwarding of sexts by young people warrants a specific and more in-depth examination using qualitative methods. Research aimed at informing strategies to prevent non-consensual sharing of sexts could be particularly valuable.

Multiple review authors identified a lack of research on the influence of social identities such as ethnicity, sexual orientation or disability on outcomes. This is a potentially important gap in knowledge, especially as the reported prevalence data suggest that involvement with sexting and/or pornography may be higher in LGBT individuals and those from ethnic minority groups [22, 25, 28, 43]. Notably, some studies have indicated that LBGT young people use pornography as a key source of information about sex, as well as to explore their sexual identity and to determine their readiness to engage in sexual activity [21, 22, 33, 44]. Research that adopts an intersectionality perspective would be beneficial for understanding the combined influence of social identities on outcomes of interest.

The current evidence base lacks geographical diversity, with the majority of findings originating from studies conducted in a small number of countries only. The extent to which findings are generalisable across countries is unclear. One review identified the extent to which a country has a liberal culture as a factor determining the existence, or extent, of gender differences in pornography use [22]. Culture as well as other country-specific factors are also likely to influence the relationship between pornography use and sexting and individual beliefs, attitudes, behaviour and wellbeing. For example, access to comprehensive, relevant and high-quality sex and relationship education.

Whilst some positive aspects to pornography and engaging in sexting were identified, the predominant focus of the studies reported across reviews, was on potential negative outcomes, or outcomes that were framed by review authors as negative. The need for more quantitative studies to adopt a wider perspective and examine the potential positives associated with pornography use for young people was highlighted in reviews by Peter and Valkenburg [22] and Koletić [23].

### Limitations

We conducted this RoR using methods that were consistent with the key principles outlined in published guidance, for example Pollock et al. 2016 [45] and 2020 [46]. This RoR is limited by the specific focus adopted in individual reviews, and the quality of reporting on primary studies and their findings by review authors. Some findings may have been omitted, selectively reported or reported inaccurately. Both pornography use and sexting are potentially sensitive issues and consequently reporting of behaviours may have been influenced by social desirability bias. Almost all of the reviews only included studies published in peer-reviewed journals and written in English, which may also have been a source of bias.

The age group of interest for this RoR was children and young people up to early adulthood, but multiple reviews included studies that had an upper age limit over nineteen years old. In addition, the reviews by both Kosenko et al. [29] and Watchirs Smith et al. [31] included at least three studies with individuals aged 18 years and older only. The wide age range of the included studies in some reviews, and the fact that data in a number of studies were derived from individuals aged 18 years and over only, are therefore potential limitations in the context of examining the experiences of children and younger adults.

We identified reviews published up to early autumn 2018, but inevitably findings were based on data obtained from earlier primary studies. Review authors did not search beyond 2017 for primary studies on sexting and 2015 for ones on pornography. Thus, data published in the last three to five years are not represented in this RoR. There may also have been reviews published since 2018 on pornography use and sexting amongst young people. However, it is extremely unlikely that any relevant reviews published in that short period of time would have significantly altered our findings and assessment of the evidence base.

We used modified DARE criteria to critically appraise included reviews and this is acknowledged as a potential limitation. The DARE criteria were not originally designed as a tool for quality assessment and have not been validated for the task. Whilst the criteria focus on a relatively small number of characteristics, reviewers were able to supplement the criteria when conducting the appraisal by recording any key observations regarding potential methodological issues or sources of bias. We incorporated these observations into the findings of the appraisal process.

## Conclusions

Evidence was identified linking both pornography use and sexting amongst young people to specific beliefs, attitudes and behaviours. However, the evidence was often inconsistent and much of it derived from cross-sectional studies, which precludes the establishment of a causal relationship. The current evidence base is also limited by other methodological issues inherent to primary studies and to reviews of these studies, as well as by key gaps in the literature, which make drawing conclusions difficult.

In the future, the use of more sophisticated and rigorous quantitative studies may help to elucidate relationships of interest. However, it is important to recognise that such research is unlikely to ever be able to determine or isolate with certainty the ‘effect’ of pornography and sexting on young people. Qualitative studies that give weight to the voices of young people themselves have an important role to play in gaining a more comprehensive and nuanced understanding of their relationship with pornography and sexting.

## Supplementary Information

**Additional file 1.** Example search strategy for MEDLINE.

## Data Availability

Not applicable.

## Abbreviations

CI: Confidence interval
DHSC: Department of Health and Social Care
LGBT: Lesbian, gay, bisexual, transgender
OR: Odds ratio
RoR: Review of reviews

## Footnotes

1 https://www.crd.york.ac.uk/crdweb/searchstrategies.asp A slightly amended version of the search filter was used for this RoR.

2 Findings from the review by Handschuh et al. included in the report to the DHSC were based on a conference abstract published in 2018. The findings reported in the current paper are based on a full journal article that the authors published on their review in 2019.
