# Supplementary material for: Pornography use and sexting amongst children and young people: a systematic overview of reviews
Source: Syst Rev. 2020 Dec 6;9:283. doi: 10.1186/s13643-020-01541-0 (PMC7720575; doi:10.1186/s13643-020-01541-0)
Supplement: Supplementary file 1 — Additional file 1. Example search strategy for MEDLINE. [file 13643_2020_1541_MOESM1_ESM.docx]

**Example search strategy for MEDLINE**

**Via OVID**

Database: Ovid MEDLINE(R) and Epub Ahead of Print, In-Process & Other Non-Indexed Citations and Daily

1 erotica/

2 porn$.ti,ab.

3 pornography.ti,ab.

4 cyberporn$.ti,ab.

5 erotica.ti,ab.

6 (sexual$ adj2 explicit$).ti,ab.

7 (sexual$ adj2 (content$ or image$ or material$ or media)).ti,ab.

8 (explicit adj2 (content$ or image$ or material$)).ti,ab.

9 sexting.ti,ab.

10 1 or 2 or 3 or 4 or 5 or 6 or 7 or 8 or 9

11 (systematic$ adj2 review$).ti,ab.

12 meta-analysis as topic/

13 meta-analytic$.ti,ab.

14 meta-analysis.ti,ab,pt.

15 metanalysis.ti,ab.

16 metaanalysis.ti,ab.

17 meta analysis.ti,ab.

18 meta-synthesis.ti,ab.

19 metasynthesis.ti,ab.

20 meta synthesis.ti,ab.

21 meta-regression.ti,ab.

22 metaregression.ti,ab.

23 meta regression.ti,ab.

24 (synthes$ adj3 literature).ti,ab.

25 (synthes$ adj3 evidence).ti,ab.

26 integrative review.ti,ab.

27 data synthesis.ti,ab.

28 (research synthesis or narrative synthesis).ti,ab.

29 (systematic study or systematic studies).ti,ab.

30 (systematic comparison$ or systematic overview$).ti,ab.

31 evidence based review.ti,ab.

32 comprehensive review.ti,ab.

33 critical review.ti,ab.

34 quantitative review.ti,ab.

35 structured review.ti,ab.

36 realist review.ti,ab.

37 realist synthesis.ti,ab.

38 pooled analysis.ti,ab.

39 or/11-38

40 review.pt.

41 medline.ab.

42 pubmed.ab.

43 cochrane.ab.

44 embase.ab.

45 cinahl.ab.

46 psyc?lit.ab.

47 psyc?info.ab.

48 (literature adj3 search$).ab.

49 (database$ adj3 search$).ab.

50 (bibliographic adj3 search$).ab.

51 (electronic adj3 search$).ab.

52 (electronic adj3 database$).ab.

53 (computeri?ed adj3 search$).ab.

54 (internet adj3 search$).ab.

55 included studies.ab.

56 (inclusion adj3 studies).ab.

57 inclusion criteria.ab.

58 selection criteria.ab.

59 predefined criteria.ab.

60 predetermined criteria.ab.

61 (assess$ adj3 (quality or validity)).ab.

62 (select$ adj3 (study or studies)).ab.

63 (data adj3 extract$).ab.

64 extracted data.ab.

65 (data adj2 abstracted).ab.

66 (data adj3 abstraction).ab.

67 published intervention$.ab.

68 ((study or studies) adj2 evaluat$).ab.

69 (intervention$ adj2 evaluat$).ab.

70 confidence interval$.ab.

71 heterogeneity.ab.

72 pooled.ab.

73 pooling.ab.

74 odds ratio$.ab.

75 (Jadad or coding).ab.

76 or/41-75

77 40 and 76

78 review.ti.

79 78 and 76

80 (review$ adj4 (papers or trials or studies or evidence or intervention$ or evaluation$)).ti,ab.

81 39 or 77 or 79 or 80

82 letter.pt.

83 editorial.pt.

84 comment.pt.

85 82 or 83 or 84

86 81 not 85

87 exp animals/ not humans/

88 86 not 87

89 10 and 88
